# MicroRNA signatures in vitreous humour and plasma of patients with exudative AMD

**DOI:** 10.18632/oncotarget.8280

**Published:** 2016-03-22

**Authors:** Catherine Ménard, Flavio A. Rezende, Khalil Miloudi, Ariel Wilson, Nicolas Tétreault, Pierre Hardy, John Paul SanGiovanni, Vincent De Guire, Przemyslaw Sapieha

**Affiliations:** ^1^ Department of Biochemistry, Maisonneuve-Rosemont Hospital Research Centre, University of Montreal, Montreal, Quebec, Canada; ^2^ Department of Ophthalmology, Maisonneuve-Rosemont Hospital Research Centre, University of Montreal, Montreal, Quebec, Canada; ^3^ Department of Engineering Physics, École Polytechnique de Montréal, Laser Processing and Plasmonics Laboratory, Montreal, Quebec, Canada; ^4^ Departement of Neuroscience, McGill University, Montreal, Quebec, Canada; ^5^ Departments of Pediatrics and Pharmacology, University of Montreal, Montreal, Quebec, Canada; ^6^ Laboratory of Membrane Biochemistry and Biophysics, Nutritional Neuroscience Section, NIAAA, NIH, Bethesda, MD, United States of America; ^7^ Department of Clinical Biochemistry, Maisonneuve-Rosemont Hospital, Quebec, Canada

**Keywords:** age-related macular degeneration, AMD, microRNAs, miRNA, biomarkers, Gerotarget

## Abstract

Age-related macular degeneration (AMD) is a leading cause of blindness worldwide affecting individuals over the age of 50. The neovascular form (NV AMD) is characterized by choroidal neovascularization (CNV) and responsible for the majority of central vision impairment. Using non-biased microRNA arrays and individual TaqMan qPCRs, we profiled miRNAs in the vitreous humour and plasma of patients with NV AMD. We identified a disease-associated increase in miR-146a and a decrease in miR-106b and miR-152 in the vitreous humour which was reproducible in plasma. Moreover, miR-146a/miR-106b ratios discriminated patients with NV AMD with an area under the Receiver Operating Characteristic curve (ROC AUC) of 0,977 in vitreous humour and 0,915 in plasma suggesting potential for a blood-based diagnostic. Furthermore, using the AMD Gene Consortium (AGC) we mapped a NV AMD-associated SNP (rs1063320) in a binding site for miR-152-3p in the HLA-G gene. The relationship between our detected miRNAs and NV AMD related genes was also investigated using gene sets derived from the Ingenuity Pathway Analysis (IPA). To our knowledge, our study is the first to correlate vitreal and plasma miRNA signatures with NV AMD, highlighting potential future worth as biomarkers and providing insight on NV AMD pathogenesis.

## INTRODUCTION

Age-related macular degeneration (AMD) is a progressive retinal pathology affecting the elderly. It is estimated that over 10 million Americans have a form of AMD and this figure is predicted to triple in the next 25 years [1, 2]. Consequently, AMD is the most important cause of vision loss in individuals over 50 and by some estimates affects more than 25% of the population over 80 years of age [3, 4]. There are two major clinical subtypes of sight-threatening AMD: dry atrophic AMD and wet exudative AMD [5]. Dry AMD is characterized by geographic atrophy while the neovascular form of AMD (NV AMD), by the growth of abnormal leaky choroidal vessels into the retina [6]. The exudative form is responsible for the majority of central vision impairment and legal blindness and accounts for 90% of clinical cases with loss of sight [1].

While genome-wide association (GWA) studies have successfully identified genetic links to AMD, there remains a void in our understanding of how extrinsic modulators of gene expression impact disease outcome. In this regard, there has been growing interest over the last decade in small non-coding RNAs called microRNAs (miRNAs) and their role as potent suppressor of gene expression in health and disease [7]. It is current thought that miRNAs control the expression of over a third of human genes and are key regulators of every primary biological process including angiogenesis, inflammation, cellular proliferation, apoptosis, differentiation, organogenesis and many more [8-11]. To date, more than 2000 of these small RNAs of about 20 nucleotides have been identified in humans and they are highly conserved throughout species [7]. Interestingly, hundreds of small RNAs have been detected in a spectrum of body fluids such as blood, urine, vitreous humour, saliva, cerebrospinal fluid and more [12, 13]. Studies have shown that in addition to being detectable in blood cells, miRNAs circulate in secreted exosomes [14, 15], micro-vesicles [16], apoptotic bodies [17], bound to the AGO2 complex [18] and in lipoproteins [19]. Much like hormones, miRNAs are secreted and provide highly specific signatures for conditions ranging from kidney disease to cancer. Moreover, SNPs that can disrupt or enhance binding of miRNAs have been reported in microRNA binding sites (miRSNPs) in specific genes and polymorphisms in miRNA binding sites are currently being assessed as potential biomarkers in oncology [20]. Interestingly, several circulating miRNAs are involved in the regulation of angiogenic and inflammatory process that are at the center of AMD pathophysiology [11, 21].

In the present study, we profiled miRNAs in the vitreous humour and plasma of patients suffering from NV AMD and control patients with non-vascular ocular pathology. We were able to identify a differential expression profile for 3 miRNAs (miR-146a-5p, miR-106b-5p, and miR-152-3p) in vitreous humour and validated this profile in circulation. We applied our findings on differential miRNA expression patterns to results from the AMD Gene Consortium (AGC) [22] and searched for NV AMD-associated DNA sequence variants resident in genomic regions encoding our three NV AMD-associated miRNAs and 484 associated binding motifs. We identified a NV AMD-associated sequence variant in *HLA-G* (rs1063320) that overlapped a genomic region with the capacity to encode a binding motif for hsa-miR-152-3p. In addition, numerous sequence variants resident in genes encoding products that are regulated by miR-146a-5p, miR-106b-5p and miR-152-3p are strongly associated with NV AMD. To our knowledge, this study is the first to identify miRNA signatures in patients suffering from NV AMD and highlights their potential future applications as diagnostic or prognostic tools.

## RESULTS

### Profiling miRNAs in vitreous humour reveals distinct expression patterns in patients with NV AMD compared to control patients with nonvascular ocular pathology

**Figure 1:**
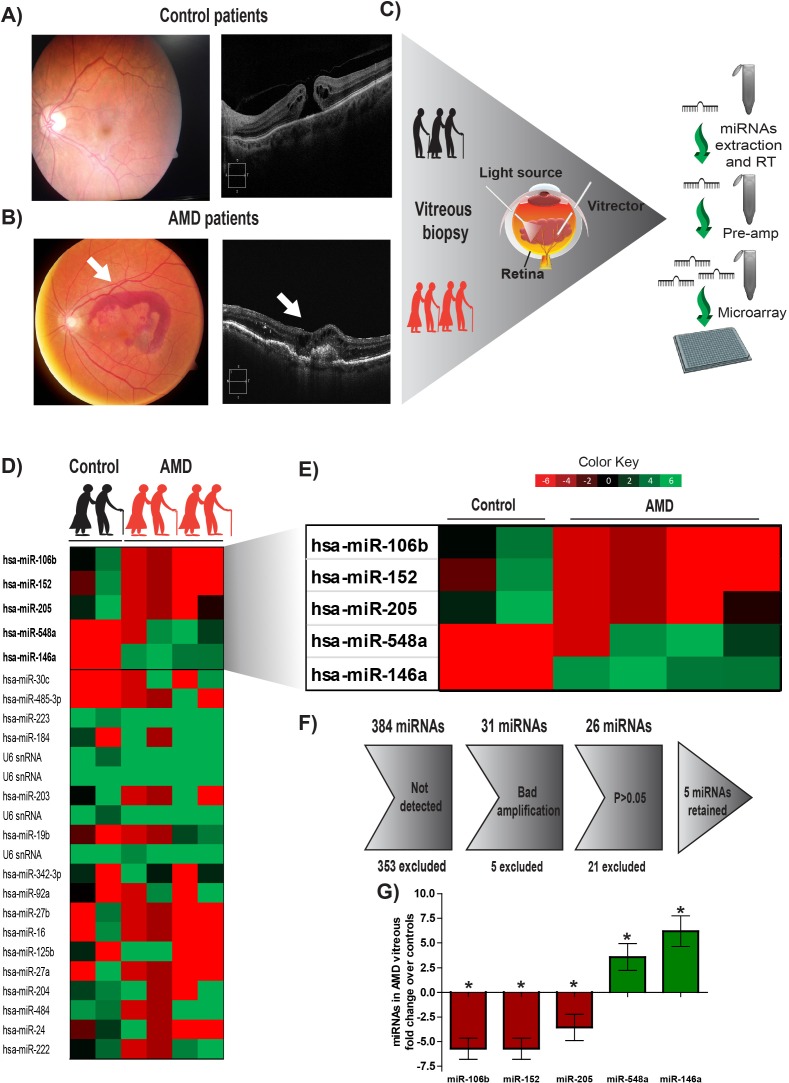
Human vitreous humour profiling identifies 5 potential miRNAs deregulated in NV AMD Vitreous humour biopsies were performed on patients with non-vascular ocular pathology (Epiretinal membrane or macular hole) **A.** or patients with exudative AMD (white arrow points to CNV) **B. C.** A work flow describing the process from patient to miRNA micro arrays. Initial pilot screen was on two samples from control patients and 4 samples from the AMD group. **D.** Complete heat map of miRNAs detected by micro arrays. **E.** Heatmap representing miRNAs identified to have a significant difference (*P* < 0.05). **F.** Gray arrow defines steps of exclusion. **G.** Histogram representation of 5 miRNAs deregulated in AMD compared to the level detected in control group (expressed as log2 (qPCR efficiency^)-ΔCt^ fold change relative to control).

All AMD patients undergoing miRNA profiling were diagnosed with pronounced CNV by Optical Coherence Tomography (OCT) (Figure 1A and 1B, right panels) and fundus image analysis (Figure 1A and 1B, left panel) before collection of vitreous humour. Patient characteristics are outlined in Tables 1 and 2. The vitreous humour of patients with nonvascular pathology (epiretinal membrane or macular hole) was used as a control. Large scale miRNA profiling using micro arrays (panel A: Applied Biosystems) was conducted on the vitreous humour of 4 patients with NV AMD and 2 control patients (work flow in Figure 1C). After analyzing individual amplification curves, 26 miRNAs had detectable amplification profiles (Figure 1D-1F). Of these, 5 miRNAs showed disease-specific expression profiles after weighted mean normalization and determination of qPCR efficiency (Figure 1E) [23, 24] where qPCR efficiencies were calculated with raw fluorescence values and gave a more precise estimation of the quantity of miRNA in the sample. This additional analysis is useful to compare qPCR reactions with similar efficiency and avoid a batch effect between different plates and qPCR reactions [25]. We applied a cut-off of 80% efficiency and excluded results under this limit. Specifically, vitreous from patients with NV AMD showed an increase in the levels of miR-548a (3.58 fold; *P*= 0.04) and miR-146a-5p (6.2 fold; *P*= 0.020) and a decrease for miR-106b (−5,73 fold; *P*= 0.010), miR-152 (−5,63 fold; *P*= 0.046) and miR-205 (−3.56 fold; *P*= 0.047) (Figure 1G) Together, these results offer novel evidence for differential miRNA expression in the vitreous of patients with exudative AMD, suggestive of either their implication in AMD pathology or being produced as a consequence of NV AMD.

**Table 1:** Patient baseline statistics

Characteristics	Control Group	Neovascular AMD
**Number of individuals**	13	13
**Mean group average; (S.D.)[ages]**	66.6 [11.2]	81.9 [9.1]
**Female Percentage (%)**	85%	69%
**OCT Values**	N.A	>250μm
**Control type number: Epiretinal membrane (ERM) Macular hole (MH)**	84	N.A.N.A.

**Table 2:** Clinical characteristics of patients having undergone vitreous biopsy

Sample	Sex	Age	Patient condition	OCT (μm)	Analysis
**C1**	F	54	NA	NA	qPCR
**C2**	F	81	MH	NA	qPCR
**C3**	F	65	MH	NA	qPCR
**C4**	F	76	MH	NA	qPCR
**C5**	F	70	ERM	NA	microarray and qPCR
**C6**	F	46	ERM	NA	qPCR
**C7**	F	84	ERM	NA	qPCR
**C8**	M	78	ERM	NA	qPCR
**C9**	F	63	CAT-MH	NA	microarray
**C10**	F	66	ERM	NA	qPCR
**C11**	F	54	ERM	NA	qPCR
**C12**	F	72	ERM	NA	qPCR
**C13**	M	57	ERM	NA	qPCR
**AMD1**	M	76	NAIVE	401	microarray
**AMD2**	M	86	NAIVE	438	microarray
**AMD3**	F	91	NAIVE	301	qPCR
**AMD4**	M	76	ACTIVE	260	microarray
**AMD5**	F	85	NAIVE	382	qPCR
**AMD6**	F	94	NAIVE	1077	qPCR
**AMD7**	M	63	NAIVE	622	qPCR
**AMD8**	F	96	NAIVE	1150	microarray and qPCR
**AMD9**	F	74	NAIVE	289	qPCR
**AMD10**	F	87	NAIVE	223	qPCR
**AMD11**	F	75	NAIVE	361	qPCR
**AMD12**	F	85	NAIVE	250	qPCR
**AMD13**	F	75	NAIVE	492	qPCR
**AMD13**	F	75	NAIVE	492	qPCR

Vitreous and plasma from a cohort of 13 patients with non-vascular retinal pathology (control group) and 13 patients with NV AMD. AMD: Age related Macular Degeneration with CNV (choroid neovascularization); naive: before anti-VEGF treatment; NA: not applicable; MH: Macular Hole; CAT: Catheter; ERM: epiretinal membrane; OCT: Optical Coherence Tomography. Analysis by microarray and/or qPCR (individual TaqMan miRNA assay).

### Validation of NV AMD-associated miRNAs in vitreous humour of larger cohorts

**Figure 2:**
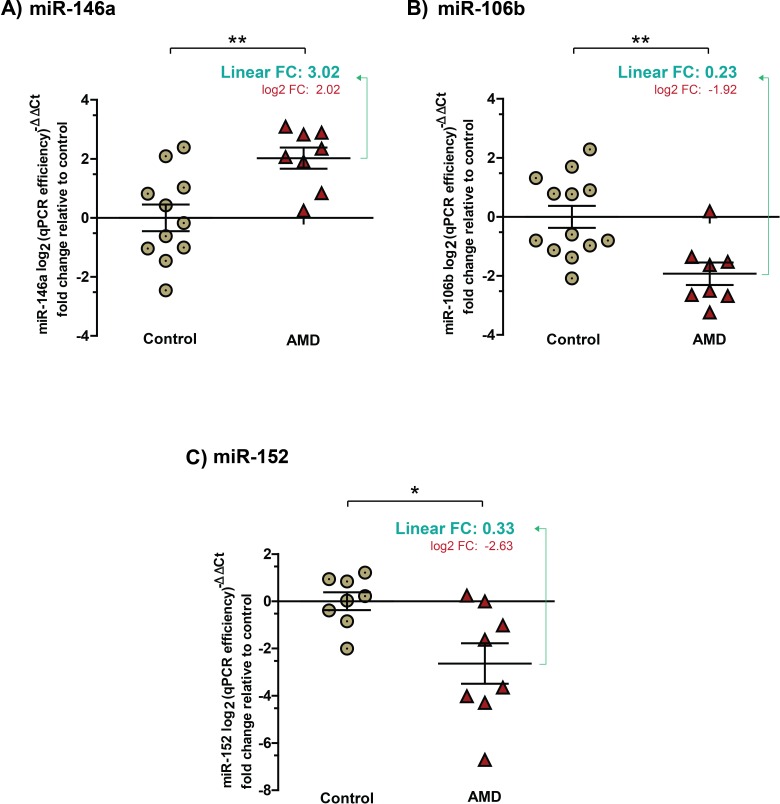
Validation of expression profiles for miR-146a, miR-106b and miR-152 in the vitreous humour of patients with NV AMD Data are represented in log2 (qPCR efficiency^)-ΔΔCt^ fold change relative to control in graphs. **A.** miR-146a increased by ∼ 3 (3.02 ± 0.5830) (corresponding log2 values: 2.02; ± 0.3587, *P* = 0.0043). **B.** Significantly decreased levels of miR-106b by ∼4 (0.23 ± 0.0797) (corresponding log2 values: −1.92 ± 0.3822, *P* = 0.003) and **C.** miR-152 by ∼ 3 (0.33 ± 0.132) (corresponding log2 values: −2.63 ± 0.8569, *P* = 0.0137) were detected in vitreous humour of patients with NV AMD.

In order to ascertain validity of the data obtained by micro arrays, we evaluated miRNA expression profiles by individual TaqMan micro in a larger cohort of patients (Table 1). As housekeeping miRNAs are not established for vitreous humour and qPCR array data did not yield a candidate, we normalized using the weighted mean of qPCRs. Results were normalized and presented as log2-transformed qPCR fold-change (Figure 2). In addition, we included corresponding fold change values in linear scale to appreciate the difference between groups.

Consistent with micro array data, individual miRNA qPCRs confirmed an increase in levels in the vitreous humour of patients with NV AMD of miR-146a (Figure 2A) by ∼ 3 (3.02 ± 0.5830), and a decrease in levels of miR-152 (Figure 2C) by ∼ 3 (0.33 ± 0.132). Levels of miR-106b also decreased (Figure 2B) by ∼4 (0.23 ± 0.0797) in NV AMD patients where this miRNA was predominantly undetected (corresponding log2 values: miR-146A, 2.02 ± 0.3587; *P* = 0.0043; miR-106b, −1.92 ± 0.3822, *P* = 0.003; miR152, −2.63 ± 0.8569, *P* = 0.0137). Conversely, the expression of miR-548a and miR-205 (both detected by micro array) did not vary significantly in the validation cohorts. Similar to array analysis, efficiency of qPCR reactions was calculated with raw fluorescence values to precisely estimate the amount of miRNAs in each sample and to compare samples with similar efficiencies [23, 24].

To determine if age was a confounding factor, we analyzed levels of miRNAs against patient age. No significant correlation was observed between individual age and the fold change of miRNAs (miR-146a r^2^ = 0.1769, miR-106b r^2^ = 0.0314 and miR-152 r^2^ = 0.1926) supporting the idea that the observed signature of vitreous humour miRNAs was attributed to NV AMD.

### AMD-specific expression of plasma miRNAs mirror levels found in vitreous humour

**Figure 3:**
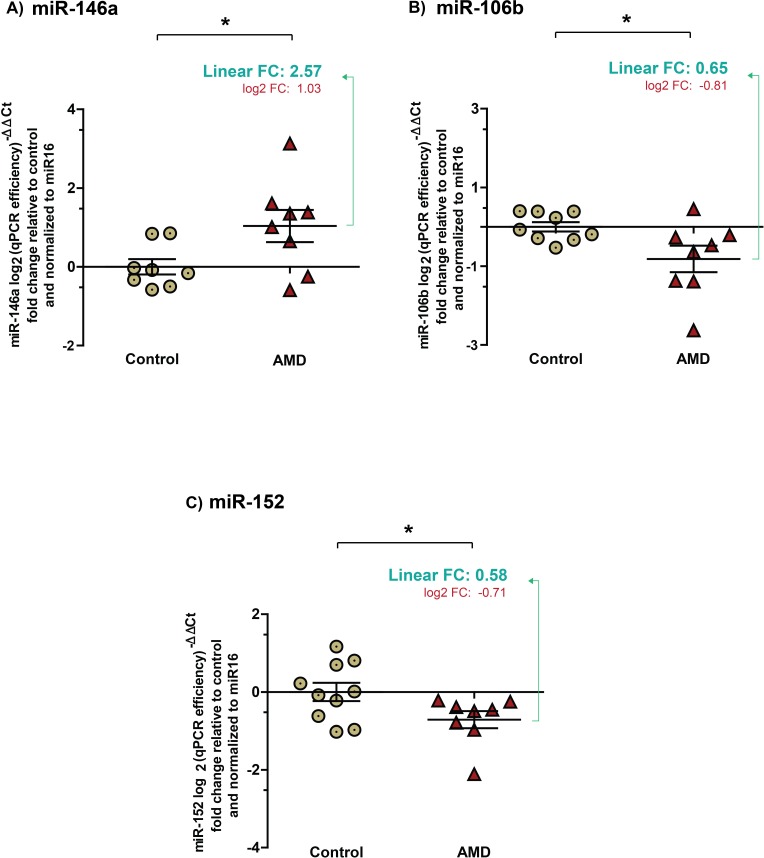
Expression profiles of miRNAs in plasma mirror levels found in vitreous humour Plasma collected from the same patients as vitreous humour profiled in figures 1 & 2. Data are represented in log2 (qPCR efficiency^)-ΔCt^ fold change relative to control in graphics **A.** A significant increase by ∼2.5 (2.57 ± 0.311) (corresponding log2 values: 1.035 ± 0.312, *P* = 0.0388) was detected for miR-146a in plasma from patients with NV AMD. Significant decreases in **B.** miR-106b by ∼1.5 (0.65 ± 0.1281) (corresponding log2 values: −0.81 ± 0.3366, *p* = 0.0301) and **C.** in miR-152 by ∼1.7 (0.58 ± 0.064) (corresponding log2 values: −0.71 ± 0.225, *P* = 0.0463) were also detected in the plasma of patients with NV AMD.

To determine if AMD-specific miRNA signatures can be obtained *via* less invasive routes, we profiled levels of miR-146a, miR-106b and miR-152 in plasma. miR-16 was used as a reference as previously described [26]. Plasma samples were tested for hemolysis and samples with an index higher than 1 were excluded (Supplemental Figure 1A). With a cutoff of hemolysis index at 1, no correlation was observed between miR-16 (Supplemental Figure 1B) and the 3 investigated miRNAs between groups (Supplemental Figure 1C-1E). As for the vitreous humour, results were normalized and presented as log2-transformed qPCR fold-change (Figure 3). Fold change values in linear scale are also presented.

Similar to that observed in vitreous humour, miR-146a was significantly increased (Figure 3A) by ∼2.5 (2.57 ± 0.311) in the plasma of patients with NV AMD while miR-106b (Figure 3B) and miR-152 (Figure 3C) were respectively decreased by ∼ 1.5 (0.65 ± 0.1281) and 1.7 (0.58 ± 0.064) (corresponding log2 values: MiR-146-a, 1.035 ± 0.312, *P* = 0.0388, miR106b, −0.81 ± 0.3366, *P* = 0.0301, miR-152, −0.71 ± 0.225*, P* = 0.0463). Similar magnitudes and expression profiles were obtained when miRNAs were normalized with weighted mean over miR-16 (Supplemental Figure 2).

Similar to the vitreous humour, no significant correlation was drawn between patient age and levels of miRNAs (miR-146a r^2^ = 0.1787, miR-106b r^2^ = 0.1564 and miR-152 r^2^ = 0.0243). Together, these results suggest that detection of plasma miRNAs mirrors profiles found in the vitreous humour and correlates with NV AMD.

### Vitreous humour or plasma ratio of miR-146a/miR-106b predicts NV AMD

**Figure 4:**
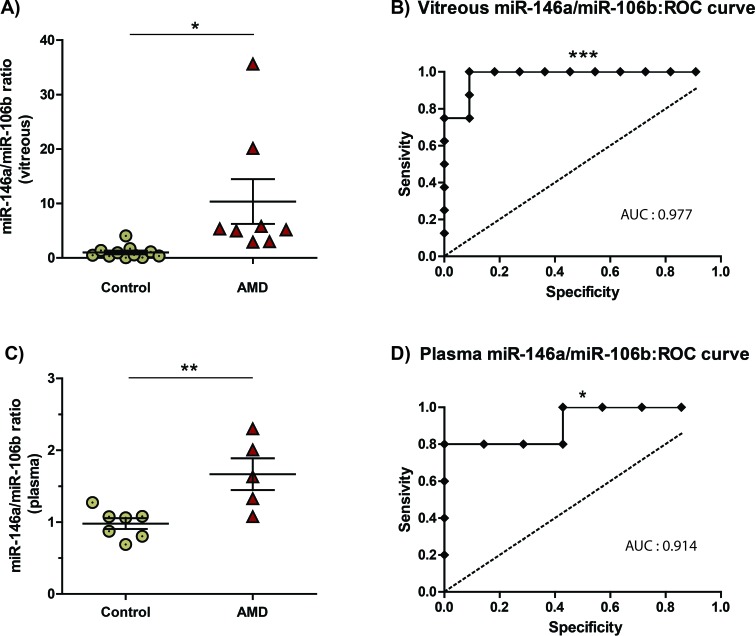
The miR-146a/miR-106b ratio in vitreous humour and blood is predictive of NV AMD MiR-146a/miR-106b fold change ratio was calculated for AMD and control for **A.** vitreous humour (10.35 ± 4.10, *P* = 0.016) and **C.** plasma (1.68 ± 0.22, *P* = 0.0072). The area under the Receiver Operating Characteristic curve (ROC AUC) for miR-146a/miR-106b ratio was B) 0,977 in the vitreous humour (*P* = 0.0005; 95% confidence interval 0.9205 to 1.034) and **B.** 0,914 in plasma (*P* = 0.0186; 95% confidence interval 0.7346 to 1.094).

Combinations of levels of different miRNAs can significantly augment the predictive value of these biomarkers [7]. We evaluated the potential of the miR-146a/miR-106b ratio to identify NV AMD patients (Figure 4). Our data demonstrated that ratios from both vitreous humour and plasma were significantly increased by ∼10 (10.35 ± 4.10, *P* = 0.016) and ∼1.7 (1.68 ± 0.22, *P* = 0.0072). We then assessed the diagnostic accuracy of these ratios as predictors of NV AMD. In this regard, Receiver Operating Characteristic (ROC) analysis is widely used to quantify how accurately a medical test can discriminate between a diseased and a non-diseased state. The resulting ROC curve is created by plotting true positive rate against false positive rate at different threshold settings. The area under the curve (AUC) then represents the probability that a random AMD patient is ranked as more likely to have AMD than a randomly chosen control patient. A maximal AUC value of 1 means that the test perfectly discriminates AMD and non-diseased patients.

The ROC AUC for miR-146a/miR-106b ratio was 0,977 in the vitreous humour (Figure 4C: *P* = 0.0005 95% confidence interval 0.9205 to 1.034) and 0,914 in plasma (Figure 4D: *P* = 0.0186; 95% confidence interval 0.7346 to 1.094). These results strongly underscore potential for plasma miR-146a/miR-106b ratios as a non-invasive biomarker for NV AMD.

### Investigation of NV AMD-associated Single Nucleotide Polymorphisms (SNPs) in miR-146a-5p, miR-106b-5p, and miR-152-3p binding sites

Dysregulation of miRNA expression is central to several human diseases. Moreover, mutations in miRNA binding sites leading to increased or decreased binding affinity can also contribute to pathophysiology. We filtered NV AMD-associated SNPs from the 77,000 person AMD Gene Consortium (AGC) cohort [22] to search for NV AMD-associated SNPs in the experimentally determined (see Methods and Table S1) target gene binding sites of miR-146a, miR-106b and miR-152. We identified a NV AMD-associated SNP in the 3′UTR of HLA-G gene, in a binding site for miR-152-3p (rs1063320). This SNP attained a *P*-value of 0.026 (*Q*-value = 0.116) for NV AMD relationship in the AGC and is found in chromosome 6 spanning nucleotide base positions 29830959 to 29830978. This finding reinforces the involvement of miR-152 in AMD pathogenesis. Further work is needed to characterize the involvement of *HLA-G* in AMD progression and the impact of this SNP on miR-152 affinity.

We next evaluated the relationship between our detected miRNAs and NV AMD related genes, using gene sets derived from the Ingenuity Pathway Analysis (IPA). The IPA Knowledge Base provides annotations on miRNA-gene relationships; we used these gene sets to filter the AGC findings on NV AMD-associated SNPs resident in genes associated with miR-146a-5p, miR-106b-5p, and miR-152-3p. Table S2 contains the gene lists and functional annotations for each miRNA.

Table S3 contains NV AMD-SNP findings for all SNPs present in each NV AMD-related gene (significant at *P* < 0.005) with a binding site for any of the 3 identified miRs. The IPA gene set includes 57 genes containing miR-146a-5p binding sites. Six of these genes (*CFH*, *NFKB1*, *LTB*, *MCPH1*, *COL13A1*, and *MMP9*) carry SNPs associated with NV AMD at *P*- values < 0.005 in the AGC - we computed *Q*-values to determine that NV AMD-associated sequence variants in each of these miR-146a-5p gene binding sequences showed an expected proportion of false positives among significant tests that was less than 2% (Table S3). There were 19 genes in the IPA gene set containing binding sites for miR-106b-5p. Of these, 1 gene (*SMAD3*) contains SNPs associated with NV AMD at *P*-values < 0.005 in the AGC cohort. *Q-values* for the strongest relationships with NV AMD were less than 0.035, indicating that the expected proportion of false positives among significant tests was less than 3.5% (Table S3). Finally, there were 10 genes with miR-152-3p binding sites in the IPA gene set. Beside the SNP in the HLA-G binding site, none of these genes in the AGC cohort contained SNPs associated with NV AMD at *P*-values < 0.005. Supplemental Figure 3 contains plots of predicted miR-146a-5p, miR-106b-5p, and miR-152-3p binding sites in genes in the chromosomal position of AGC findings on NV AMD.

## DISCUSSION

The prevalence of AMD in developed countries will continue to rise dramatically with the aging population [4, 6, 27]. To our knowledge, the present study is the first to profile miRNAs in human vitreous humour from patients with NV AMD. We used a bilateral approach where we first investigated miRNA signatures in vitreous humour and correlated the identified miRNAs with plasma levels in corresponding patients. Specific miRNA signatures in vitreous humour of patients with NV AMD lead us to identify three miRNAs (miR-146a, miR-106b and miR-152) that vary with disease (not with age) and have described roles in inflammation and angiogenesis. Secondly, using a GWA study we identified miRSNPs located in binding sites in genes associated with CNV phenotypes in AMD for these three miRNAs. DNA sequence variants found in miRNA binding sites could increase or decrease their binding and hence influence their function as translation regulators.

The presence of miRNAs in human vitreous humour may be important both in further understanding AMD pathogenesis and may provide insight on systemic biomarkers for AMD diagnostic. Of the 3 miRNAs identified, only one (miR-146a) was significantly increased and 2 decreased (miR-106b and miR-152) in NV AMD. Our results suggest a 3.0 fold increase in miR-146a in the vitreous humour of patients with NV AMD. In line with our findings, Lukiw et al. showed a 3-fold increase of miR-146a in the retina of patients with AMD [28]. Induction of miR-146a may be a protective mechanism that dampens innate immunity [29, 30]. MiR-146a has been reported to be induced by lipopolysaccharide (LPS), as well as by inflammatory cytokines IL-1β and TNFα [29, 31, 32] and has the ability to influence inflammatory cascades by interfering with IRAK and TRAF-6 [29, 33] as well as by directly down-regulating IL-6 [34]. Both IL-1β and IL-6 are associated with progression of CNV [33, 35], hence, miR-146a may be a protective regulator of inflammation in NV AMD. Finally, it is notable that the mature hsa-miR-146a-5p miRNA has been shown to bind the CFH gene in human neural [36, 37] and vascular endothelial cells [38].

Conversely, the detected reduction of miR-106b and miR-152 in the vitreous humour may exacerbate CNV given that miR-106b can decrease VEGF-A expression [39, 40]. In this line, a recent study implicated miR-106b in post-ischemic neovascularization in mice [41] *via* miR-106b-dependant regulation of IL-8 [42, 43]. Similarly, the significant decrease of miR-152 may aggravate CNV by allowing angiogenic factors involved in AMD such as Fibroblast growth factor 2 (FGF2) to rise [44]. The tandem dysregulation of miR-106b and miR-152 could therefore contribute to increasing the burden of pro-angiogenic factors such as VEGF, FGF2 and IL-8 in the vitreous humour/retina, yielding an environment conducive to propagation of CNV [45].

Noninvasive biomarkers are still lacking for early detection of AMD. Profiling of miRNAs in body fluids such as blood shows promises for diagnostic, follow up and prognostic of human diseases [10]. Having for main goal to identify circulating miRNAs specific to AMD, we chose to first profile vitreous humour of patients with AMD. While Grassman et al. and Szermraj et al. both reported AMD-specific miRNA profiles in blood; our study was designed to correlate vitreous humour and blood miRNAs [46, 47]. When compared together, best hits for AMD-specific miRNAs in blood do not replicate between studies. However, it is worth mentioning that Szemraj et al. also reported an increase for miR-146a in the blood of their AMD cohort. Our profiling strategy initially called for identification of prospective miRNAs in vitreous humour and then used different analytical strategies that can potentially explain discrepancies between studies. This includes the matrix used, methods of RNA isolation, large scale profiling technologies and more [10]. In order to usefully translate our finding to a diagnostic test, signatures of circulating miRNAs must be established early in the disease process or ideally prior to onset. A non-invasive and predictive marker could be of interest considering the fact that lifestyle modifications (diet, smoking, obesity, etc.) can influence development of AMD. Future studies will need to be designed to address this point.

To further investigate the potential involvement of miR-146a, miR-106b and miR-152 in AMD pathogenesis, we used experimentally validated miRNA targets to filter specific NV AMD associated loci in the AGC. The value of our findings on the residence of a NV-AMD-associated SNP in a hsa-miR-152-3p target-encoding site is to guide future work, with a possible focus on HLA-G. However, the 3 million AGC probe feature set contained less than 5% of sequence variants within putative miR-146a-5p, miR-106b-5p and miR-152-3p predicted binding sites limiting the coverage of our analysis. To overcome this limitation, we further evaluated the relationship between our detected miRNAs and NV AMD related genes, using gene sets derived from the Ingenuity Pathway Analysis (IPA). Interestingly, 10% of hsa-miR-146a-5p targets also had AMD associated sequence variants with *P*-values < 0.005 and false discovery rates < 1.5%.

Our study provides the first evidence that a specific miRNA signature is found both in the vitreous humour and plasma of patients with exudative AMD. As such, it offers proof-of-concept that miRNAs in systemic circulation may potentially be biomarkers for AMD and provides novel insight into miRNA-driven pathogenesis. Moreover, analysis of specificity and sensitivity (ROC curves) of the ratio between miR146a/106b suggests their potential for plasma-based biomarkers of NV AMD. In addition, detection of SNPs located in miRNA binding sites may be predictive of disease development. From a therapeutic perspective, miRNAs are endogenous multi-target molecules and hence can be harnessed to modify disease outcome. They fill the growing interest in drug design for multi-target drugs. Generating synthetic therapeutic miRNAs or antagomirs is cost-effective and easy to produce and clinical trials are currently underway using inhibitors or synthetic mimics of miRNAs [21, 48]. Therapeutic miRNAs may also influence the effects of prospective treatments for AMD such as Rapamycin (inhibitor of mTOR) [49] which has been suggested to decrease symptoms of AMD [50-53]. Taken together, identifying specific miRNA biomarkers for AMD would provide an invaluable tool for early intervention and disease modification. Therapeutic modulation of miRNAs may provide an alternative and may significantly reduce costs and toxicity of current anti-VEGF biologics and offer new therapeutic avenues to supplement the current options used to manage CNV.

## MATERIALS AND METHODS

### Vitreous humour sample and blood collection

All patients previously diagnosed with Wet AMD were followed and operated by a single vitreoretinal surgeon (F.A. Rezende). Control patients underwent surgical treatment for nonvascular pathology (epiretinal membrane or macular hole) by the same surgeon. In an operating room setting, patients underwent surgery under local retro/peribulbar anesthesia. A 5% povidone-iodine solution was used to clean the periocular skin, and topical instillation into the eye and within the cul-de-sac was left in place for 5 minutes. Three-port25-gauge trans conjunctival *pars plana* vitrectomy was performed through 25-gauge valved cannulas (Alcon). Under microscope visualization using a wide-angle viewing system (Resight, Zeiss), undiluted vitreous humour was collected with a 25-gauge vitrector. Vitreous humour was aliquoted in sterilized tubes and directly frozen on dry ice. Blood collection had been proceeding before solutes (IV) installation and vitreous humour biopsy. Blood was collected in tube containing 7.2 mg of K2 EDTA tube (purple). To collect plasma, blood was centrifuged for 10 min at 1500 X g RPM. The plasma was transferred in new tubes and immediately frozen at −807°C.

### miRNA array and microRNA extraction

Total RNA was isolated from vitreous humour samples by using TRIzol^®^ Reagent (Invitrogen) in accordance with the manufacturer's instructions. Equal volumes of 50μl of vitreous humour were used for miRNA extraction using 50μl of TRIzol^®^ and 1μl of 20mg/ml of glycogen was added to increase the extraction specificity. Total RNA was eluted in 20μl of DEPC water. Total RNA samples of 2 controls and 4 wet AMD patients were shipped to IRIC's genomic platform (Université de Montréal) to process the Human MicroRNA Card A v2 (Life Technologies). Retrotranscription (RT) was processed with the Megaplex™ RT for TaqMan^®^ MicroRNA Assays (Human Pool A), followed by microRNA pre-amplification with the Megaplex™ PreAmp primers kit for TaqMan^®^ MicroRNA Assays (Human Pool A) and the qPCR array assay (Human Card A including detection of 384 microRNAs and endogenous controls). A plate with ultrapure water was processed as negative control for primer dimerization detetection.

### Individual qPCR validation

MicroRNAs from 50μl of vitreous humour or plasma were extracted with the TaqMan^®^ miRNA ABC magnetic kit (Anti-miRNA Bead Capture for Human Panel A) by following instructions supplied by Life Technologies. Elution was done in 50μl and 100μl for vitreous humour and plasma respectively. RT and pre-amplifications were processed as indicated in the protocol for creating custom RT and pre-amplification pools using 20X TaqMan^®^ MicroRNA Assays from Life Technologies. 5μl of RT and qPCR primers from each TaqMan^®^ MicroRNA Assays (miR-205, miR-106b, miR-146a, miR-152, and miR-548a) were pooled in a total volume of 500μl TE buffer pH 8.0. 3μl of extracted microRNA was used to for the TaqMan^®^ MicroRNA Reverse Transcription Kit (Life Technologies), 2.5μl of RT product was used for pre-amplification was assessed with 12 cycles as recommended in the Life Technologies protocol. Total volume (25μl) of pre-amplification product was diluted with 175μl of 0.1X TE buffer, pH 8.0. 2μl of the dilution was used as template in a total volume of 10μl of 20X TaqMan^®^ MicroRNA Assay for each miRNA identified in the array (miR-205, miR-106b, miR-146a, miR-152, and miR-548a). The same strategy was were processed plasma samples. qPCRs were processed 3 to 4 times to avoid technical variation.

### Normalization and statistical analysis

Realtime PCR gives expression levels of the gene/miR as a threshold cycle (Ct) (inversely proportional to relative expression of target gene/miR). According to Taqman protocol, 40 cycles were considered as the detection threshold, above which miRs were considered as undetectable.

In order to normalize miRNA data and calculate fold changes of each miRNA, ΔCt values were calculated by subtracting the Ct value of the endogenous control from the Ct of the target. However, except for plasma samples where miR16 is recognized as an endogenous control, at present there is no consensus concerning suitable miR endogenous control miR. To minimize systematic technical or experimental variation in vitreous humour samples, we opted here for a method considering all miR (miR16, miR106b, miR146a and miR152) as endogenous controls, also called weighted normalization, as follow:

$${\text{Ct}}_{0}=\sum_{j}^{n}\text{Ctj }x\;\frac{{\left(\frac{1}{\text{STD}\left(\text{Ctj}\right)}\right)}^{\text{wmp}}}{\sum_{i=1}^{n}\frac{1}{\text{STD}\left(\text{Cti}\right)}},$$

with *n* corresponding to the number of genes (miRNAs), *j* to miRNAs, *i* to sample and *wmp* to the weighted mean power.

Technically, this miR Ct_0_ average was used as endogenous reference, for each sample.

$$\Delta \text{Ct}={\text{Ct}}_{\text{miR}}-{\text{Ct}}_{0}$$

The ΔΔCt was then calculated by subtracting the ΔCt average of control patients from AMD patient.

$$\Delta \Delta \text{Ct}=\Delta {\text{Ct}}_{\text{AMD}}-\Delta {\text{Ct}}_{\text{control}}$$

Fold change (FC) was calculated using the following formula 2^−ΔΔCT^, where the value 2 represents a theorical micro efficiency of 100%. For more accuracy, we evaluated micro array and TaqMan individual qPCR efficiency with free online software QPCR, which calulated 85% for the qPCR array (1,7) and 88% for indivivual qPCR (1,75).

$${\text{FC}=\text{Efficiency}}^{-\Delta \Delta \text{Ct}}$$

For performing T-statistics, variance should be normally distributed (bell curve); a log2 transformation was then applied to fold change values.

Log-transformed data = Log (Fold change; 2)

Log-transformed data were finally median-centered (average log-transformed controls then equaled 0).

Graphical data = Log-transformed data - mean (Log-transformed control)

Student T-test was used to statistically compare the fold change value of each group, with a and *p* < 0.05 considered as significantly different. All samples showing efficiency with less than 80% of amplification, weak amplification curves, with clinical issues (ex: glaucoma, eye medical treatment,) or outliers (http://graphpad.com/quickcalcs/Grubbs1.cfm) were excluded of the analysis.

### miR-associated SNP analysis

#### Data sources & subjects

We used public-access results from the National Eye Institute-supported AMD Gene Consortium (AGC) [22] to address our aims. In 2013 the AGC published findings from a large-scale multi-center genome-wide study on the molecular genetics of AMD (http://nih.gov/news/health/mar2013/nei-03.htm). All AGC research was conducted according to the principles expressed in the *Declaration of Helsinki* and was initiated only after approval by institutional review boards (IRB) in each of the 18 participating centers. Informed consent was obtained from all participants. Details of human subject procedures, IRB approvals, and references to consenting processes exist at:http://www.nature.com/ng/journal/v45/n4/extref/ng.2578-S1.pdf—pp. 30–41. All AGC data have been de-identified.

The analytic sample contained > 4450 people with NV AMD and > 40,000 age- and sex-matched AMD-free controls of European and Asian ancestry. All findings passed quality assessment tests for completeness of genotyping and Hardy-Weinberg equilibrium. Details on methods and quality control procedures exist in Fritsche *et al.*[22]

#### Regions of interest

Gene annotations and positional coordinates for genes encoding hsa-miR-146a-5p, hsa-miR-106b-5p, hsa-miR-152-3p, and their target motifs were obtained with the Ensembl 80 database (21 March 2015, GRCh38.p2,http://useast.ensembl.org/index.html) *via* an interface to TarBase7 (http://diana.imis.athena-innovation.gr/DianaTools/) in the Regulation 80 utility. Lists of genes encoding miRNA targets that have been validated with luciferase reporter assays, qPCR, and Western blots were assembled for each of the 3 miRNAs from TarBase 7 (http://diana.imis.athena-innovation.gr/DianaTools/index.php?r = tarbase/index) and miRTarBase (http://mirtarbase.mbc.nctu.edu.tw/) - in some cases positional coordinates of miRNA motif-encoding areas were not available. Computational evidence on location of hsa-miR-146a-5p, hsa-miR-106b-5p, and hsa-miR-152-3p motif-encoding regions was obtained with microT-CDS ((http://diana.imis.athena-innovation.gr/DianaTools/), applying a threshold of greater > 0.70 for strong prediction) and miRDB ((http://mirdb.org/miRDB/), applying a Target Score threshold > 80). We used HaploReg v3 (http://www.broadinstitute.org/mammals/haploreg/haploreg_v3.php) with data from the 1000 Genomes Project to identify co-inherited loci (R^2^ > 0.80, EUR cohort as reference) as valid proxy measures in cases that SNPs in miRNA target motif regions were not present in the gene chip feature set reported by AGC. The Ingenuity Pathway Analysis knowledge base (June 2015 version) was used to identify genes in which DNA sequence or DNA sequence products have been implicated to affect or to be affected by hsa-miR-146a-5p, hsa-miR-106b-5p, or hsa-miR-152-3p.

#### Data analysis

In Phase 1 we annotated and enumerated aspects of genomic regions of interest - these included positional coordinates of motif-encoding regions, resident genes, and DNA sequence variants within the encoding regions. The next step (Phase 2) was to assess putative differences in allelic frequencies in miRNA target-encoding loci between people with NV AMD and their age- and sex-matched peers. The AGC probe set was not optimized for such purposes and thus we elected to also examine findings in all other loci in target-encoding genes (Phase 3) for the purpose of determining which NV AMD-associated genes may be the most promising candidates for focused work on miRNA target-encoding regions.

NV AMD-gene relationships were analyzed in Phases 2 and 3 from AGC data using findings from additive models adjusted for population stratification.[22] In Phase 2, inferences were based an alpha-level of 0.05 and adjusted by the number of tested SNPs in each motif-encoding region (*P* = 0.05/(N)^1/2^). In Phase 3 we evaluated findings in regions of interest with Bonferroni-adjusted *P*-values and *Q*-values (*Q*-values were computed with *QValue* software package (www.genomics.princeton.edu/storeylab/qvalue)).

## SUPPLEMENTARY MATERIALS FIGURES AND TABLES








