# Supplementary material for: MicroRNA signatures in vitreous humour and plasma of patients with exudative AMD
Source: Oncotarget. 2016 Mar 22;7(15):19171–84. doi: 10.18632/oncotarget.8280 (PMC4991373; doi:10.18632/oncotarget.8280)
Supplement: Supplementary file 1 [file oncotarget-07-19171-s001.pdf]

MicroRNA signatures in vitreous humour and plasma of patients with exudative AMD

Supplementary Materials

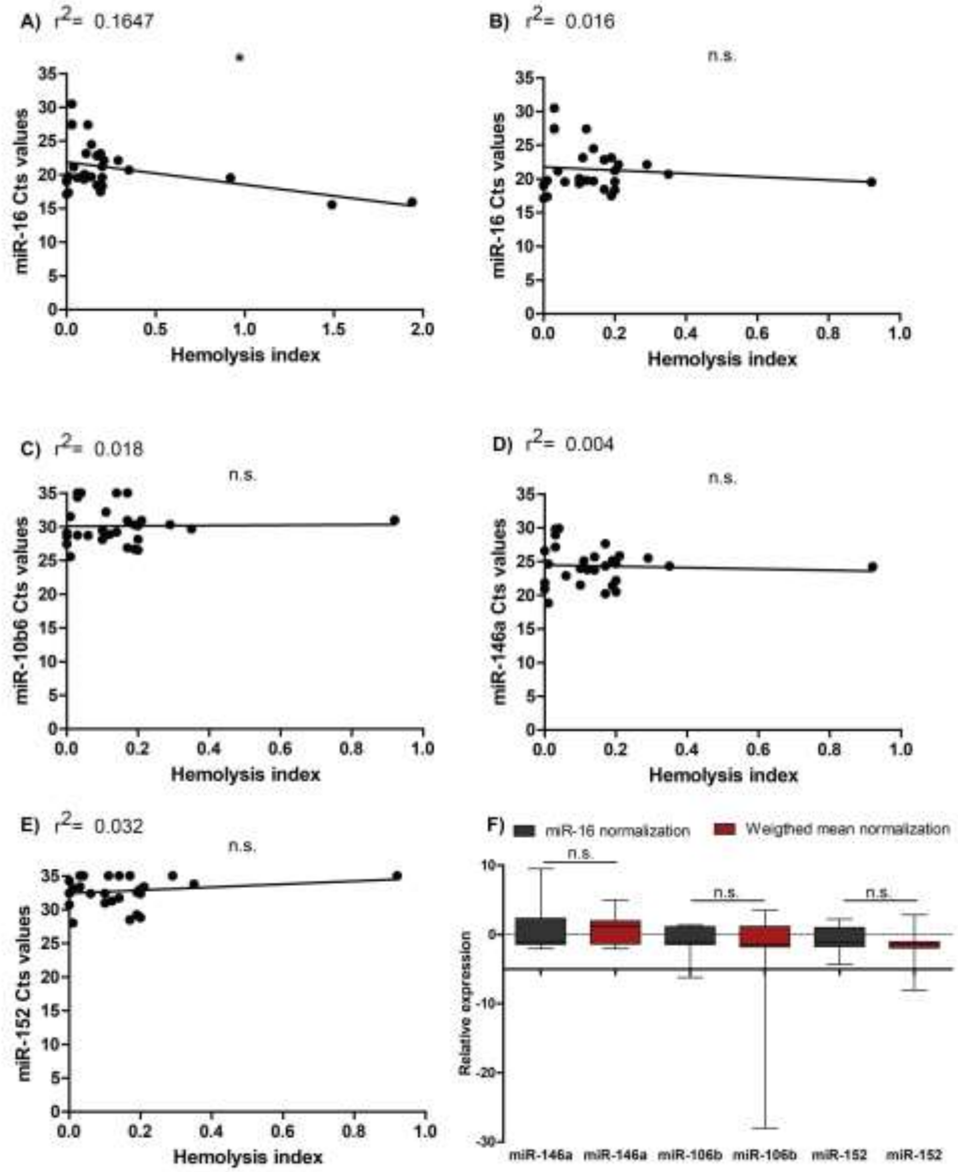

Supplemental Figure 1: Hemolysis index in correlation with raw CT values and 695 comparison of normalization strategies. **A.** miR-16 raw CT values without the cutoff 696 of the hemolysis index at 1. A significant negative correlation (P=0.0235) is obtained with a r2 697 =0.1647. **B.** When the cutoff is set for samples with a hemolysis index > 1, no 698 significant correlation is observed for miR-16. **C.**, **D.** and **E**) No correlation is found 699 between raw CT detection and hemolysis index for miR-146a, miR-106b and miR-152 700 respectively. **F.** Comparison of both normalization strategies miR-16 (gray) vs weighted 701 mean (red) including relative expression for all samples. No significant difference is 702 observed between normalization strategies.

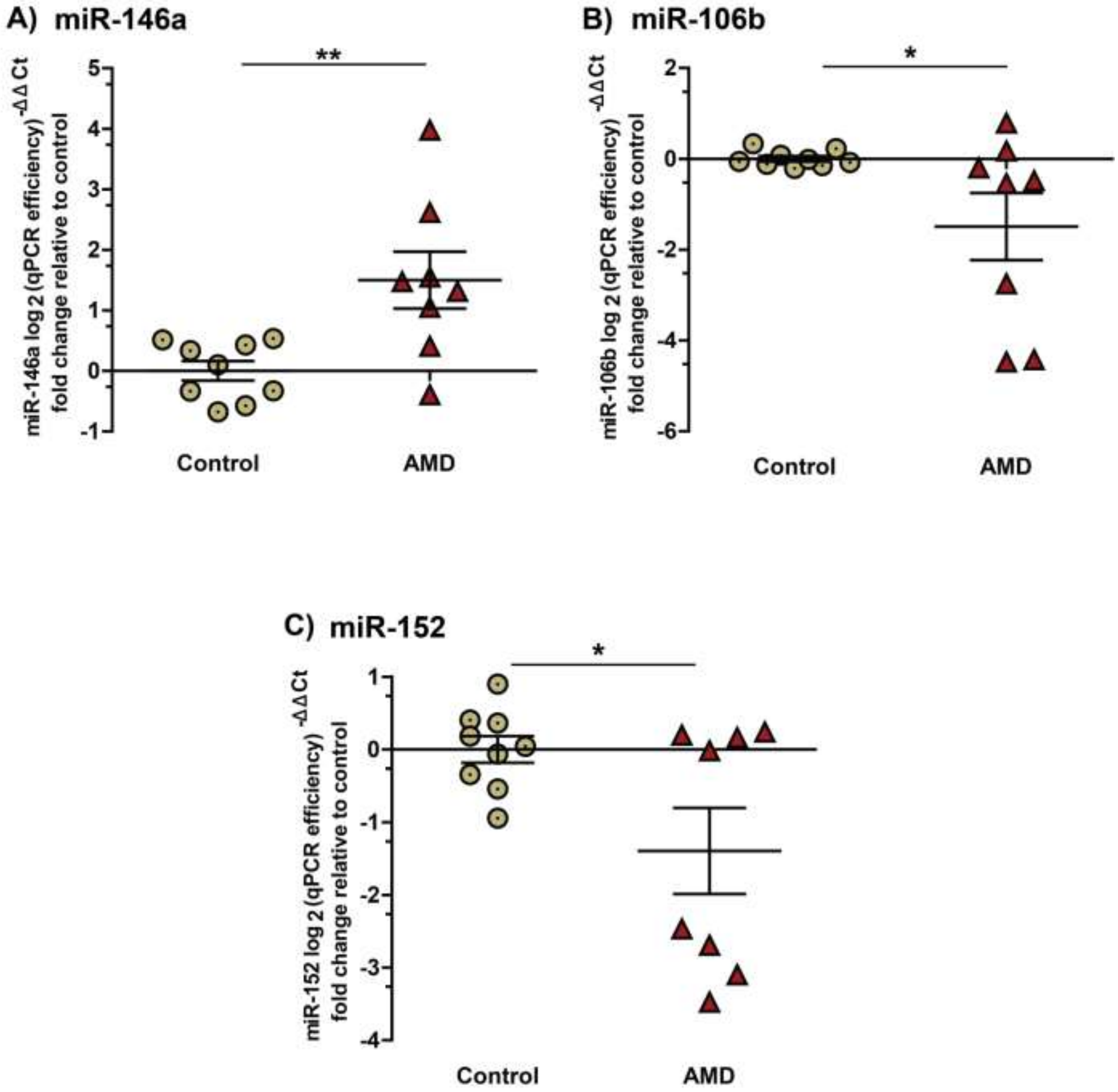

Supplemental Figure 2: Normalization of plasma miRNAs by weighted mean conserves expression profiles. Plasma was collected from the same patients as the vitreous humour profiled in figures 1 & 2. Data are represented in $\log_2$ (qPCR efficiency$)^{-\Delta\Delta Ct}$ fold change relative to control in graphics **A.** A significant increase of ~1.5 (1.500 $\pm$ 0.4705, P = 0.0064) was detected for miR-146a in plasma from patients with NV AMD. Significant decreases in **B.** miR-106b by ~1.5 (-1.489 $\pm$ 0.7397, P = 0.0496) and **C.** in miR-152 by ~1.5 (-1.394 $\pm$ 0.5916, p = 0.0318) was detected in the plasma of patients with NV AMD.

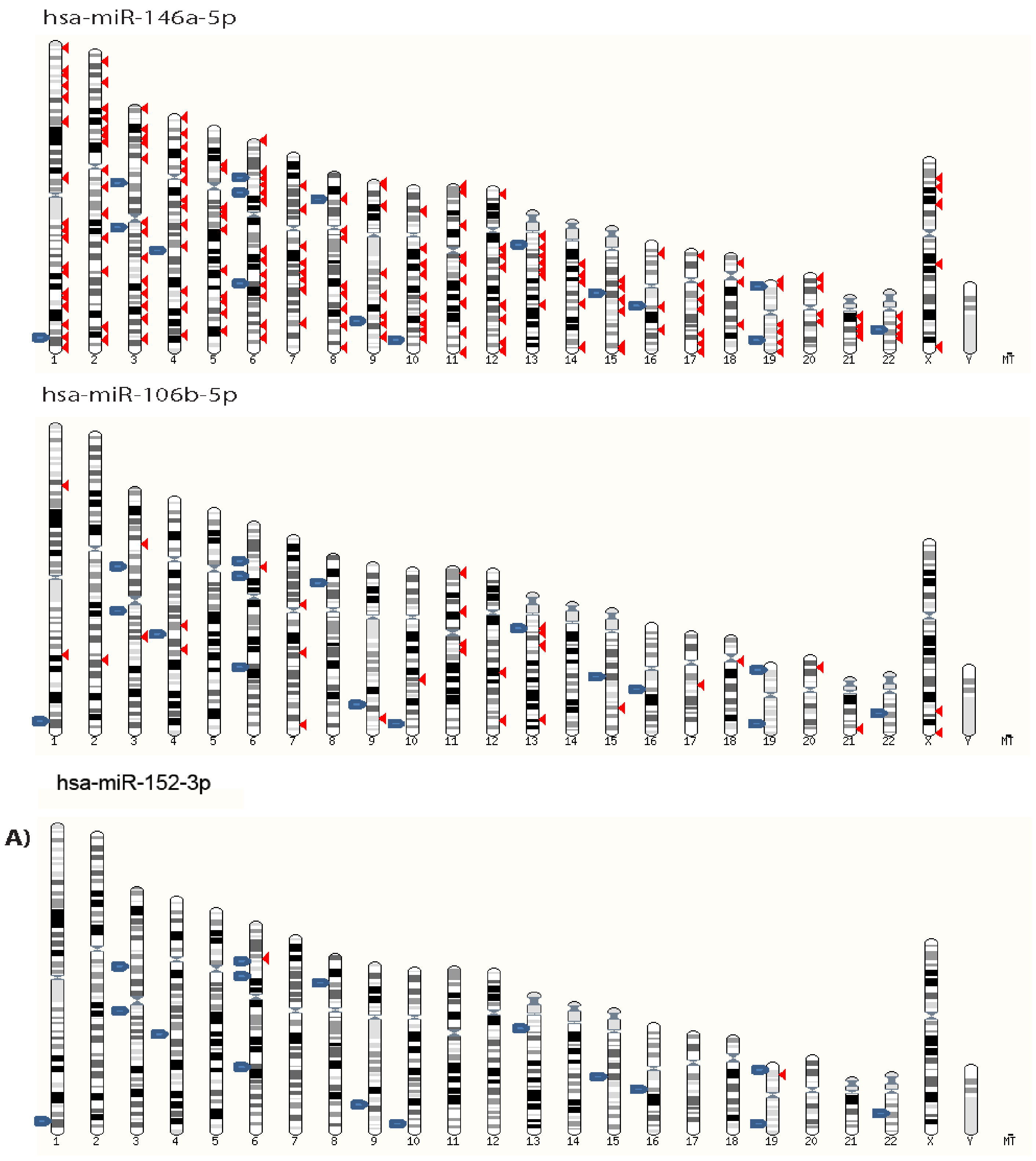

Supplemental Figure 3: **Loci encoding putative hsa-miR-146a-5p, hsa-miR-106b-5p, and hsa-miR-152-3p target motifs**. Red arrows indicate position of miRNA loci within chromosomes, blue arrows indicate locations of SNPs attaining genome-wide statistical significance ($P < 5 \times 10^{-7}$) for association with AMD. Positional coordinates of pertinent regions were obtained with the Ensembl 80 database (21 March 2015, GRCh38.p2).
